# IGF-I Influences Everolimus Activity in Medullary Thyroid Carcinoma

**DOI:** 10.3389/fendo.2015.00063

**Published:** 2015-05-05

**Authors:** Erica Gentilin, Carmelina Di Pasquale, Martina Rossi, Federico Tagliati, Teresa Gagliano, Roberta Rossi, Mariarosa Pelizzo, Isabella Merante Boschin, Ettore C. degli Uberti, Maria Chiara Zatelli

**Affiliations:** ^1^Section of Endocrinology and Internal Medicine, Department of Medical Sciences, University of Ferrara, Ferrara, Italy; ^2^Laboratorio in rete del Tecnopolo “Tecnologie delle Terapie Avanzate” (LTTA), University of Ferrara, Ferrara, Italy; ^3^Department of Surgical, Oncological and Gastroenterological Sciences, University of Padova, Padova, Italy

**Keywords:** IGF-I, medullary thyroid carcinoma, everolimus, mTOR, calcitonin

## Abstract

**Context:**

Medullary thyroid carcinoma (MTC) is a rare tumor originating from thyroid parafollicular C cells. It has been previously demonstrated that insulin-like growth factor I (IGF-I) protects MTC from the effects of antiproliferative drugs. Everolimus, an mTOR inhibitor, has shown potent antiproliferative effects in a human MTC cell line, TT, and in two human MTC primary cultures.

**Objective:**

To verify whether IGF-I may influence the effects of everolimus in a group of human MTC primary cultures.

**Design:**

We collected 18 MTCs that were dispersed in primary cultures, treated without or with 10 nM–1 μM everolimus and/or 50 nM IGF-I. Cell viability was evaluated after 48 h, and calcitonin (CT) secretion was assessed after a 6 h incubation. IGF-I receptor downstream signaling protein expression profile was also investigated.

**Results:**

Everolimus significantly reduced cell viability in eight MTC [by ~20%; *P* < 0.01 vs. control; everolimus-responders (E-R) MTCs], while cell viability did not change in 10 MTCs [everolimus-non-responders (E-NR) MTCs]. In E-R MTCs, IGF-I blocked the antiproliferative effects of everolimus that did not affect CT secretion, but blocked the stimulatory effects of IGF-I on this parameter. IGF-I receptor downstream signaling proteins were expressed at higher levels in E-NR MTC as compared to E-R MTCs.

**Conclusion:**

IGF-I protects a subset of MTC primary cultures from the antiproliferative effects of everolimus and stimulates CT secretion by an mTOR mediated pathway that, in turn, may represent a therapeutic target in the treatment of aggressive MTCs.

## Introduction

Insulin-like growth factor I (IGF-I) system has been described in both rat (1) and human medullary thyroid carcinoma (MTC) (2), where it stimulates DNA synthesis (3). In a human MTC cell line, the TT cells, IGF-I increases cell proliferation, DNA synthesis, and the percentage of cells in S phase, all effects counteracted by co-incubation with either an IGF-I antibody or an IGF-I receptor (IGF-I R) antibody (4). In addition, it has been previously demonstrated that IGF-I protects TT cells from drug-induced cytotoxicity (5). Therefore, IGF-I may hamper the effects of antiproliferative drugs in MTC.

New medical approaches became lately available for MTC, thanks to the development of several agents that target tyrosine kinases. Clinical trials have reported variable results, since patients may develop drug resistance with consequent tumor progression (6). An alternative medical approach for neuroendocrine tumors, including MTC, is represented by mTOR inhibitors, such as everolimus. The latter displays antiproliferative effects on a wide spectrum of tumors including neuroendocrine tumors, both *in vitro* and *in vivo* (7–11). Moreover, it has been recently demonstrated that everolimus inhibits cell viability in a dose- and time-dependent fashion and reduces mTOR phosphorylation in a human MTC cell line and in two human MTC primary cultures (12). In addition, everolimus treatment of two patients with progressive metastatic MTC was associated with disease stabilization in one and disease progression in the other patient (13), indicating that mTOR pathway may effectively control MTC cell proliferation in a subset of patients. The variable effects of mTOR inhibitors may be ascribed to independent signaling mechanisms, activated by several growth factors, including IGF-I.

The aim of our study is therefore to verify whether IGF-I may influence the effects of everolimus in a group of human MTC primary cultures.

## Materials and Methods

### Materials

All reagents, if not otherwise specified, were purchased from Sigma-Aldrich (Milano, Italy). Everolimus was provided by Novartis Pharma (Basel, Switzerland).

### Human MTCs

The samples derived from 18 patients diagnosed and operated on for MTC at the Section of Endocrinology and Internal Medicine of the University of Ferrara, and at the Department of Surgical, Oncological and Gastroenterological Sciences of the University of Padova. Table 1 shows patients’ characteristics and pre-operative hormonal values. All patients (six males and 12 females; age = 52.1 ± 3.9 years) underwent total thyroidectomy with central neck lymph node dissection and had histological and immunohistochemical diagnosis of MTC.

**Table 1:** **MTC patients clinical characteristics**.

No.	Sex	Age	Plasma CT (pg/ml)	Stage	Inheritance
1	M	46	157	II	SP
2	M	40	1500	II	FMTC
3	M	47	940	II	MEN2A
4	M	39	1500	II	SP
5	F	35	700	II	SP
6	F	32	28	II	MEN2A
7	F	35	74	I	SP
8	F	33	19	I	FMTC
9	F	42	153	II	SP
10	M	52	207	II	SP
11	F	44	2350	II	SP
12	M	56	1258	II	SP
13	F	79	1500	III	SP
14	F	71	2578	III	SP
15	F	73	3848	IVc	SP
16	F	75	1500	III	SP
17	F	69	3405	III	SP
18	F	70	9227	IVb	SP

*FMTC, familial medullary thyroid carcinoma; MEN2A, MEN2A RET mutation; SP, sporadic disease*.

### Tissue collection and primary culture

Tissues were collected following the guidelines of the local committee on human research, and immediately minced in RPMI 1640 medium under sterile conditions. Primary cultures were then prepared are described previously (14, 15). Cells were then treated with test substances, with further evaluation of hormone secretion and cell viability. Informed consent of the patients was obtained for disclosing clinical investigation and performing the *in vitro* study.

### Cell viability

The effects of everolimus and IGF-I on MTC cell viability *in vitro* were assessed by ATPlite assay (Perkin-Elmer, Monza, Italy) on the Wallac Victor™ 1420 Multilabel Counter (Perkin-Elmer) as previously described (16). Cells were treated after 24 h with or without 10 nM–1 μM everolimus and/or 50 nM IGF-I. Treatments were renewed after the first 24 h of incubation. Cell viability was assessed after 48 h. Results were obtained by determining the mean value of six replicates.

### Protein expression panel

Tissues were dissolved in cell lysis buffer (Bio-Rad, Milano, Italy) supplemented with cell lysis factor QG (Bio-Rad, Milano, Italy) and 2 mM phenylmethanesulfonylfluoride. Protein concentration was measured by BCA Protein Assay Reagent Kit (Pierce, Rockford, IL, USA), as previously described (17). Bio-plex^®^/Luminex^®^ Technology (Bioclarma Research and Molecular Diagnostics, Torino, Italy) was employed to assess total protein levels of IGF-I receptor (IGF-I R), AKT, p70S6K, p38MAPK, ERK1/2, and CREB in MTC tissue samples. The following phosphorylated forms were also investigated: p(Tyr1131) IGF-I R, p(Ser473) AKT, p(Thr421/Ser424) p70S6K, p(Thr180/Tyr182) p38MAPK, p(Thr202/Thr204,Thr185/Thr187) ERK1/2, and p(Ser133) CREB levels.

### Calcitonin assay

Calcitonin (CT) was measured in conditioned medium from primary cultured cells by an ELISA kit (DRG, Springfield, NJ, USA), after a 6 h treatment without or with the test substances. The intra- and inter-assay variation coefficients were 2.8–5.7% and 6.1–7.4%, respectively. The detection limit was 1.0 pg/ml. Assays were performed in duplicate.

### Statistical analysis

Fisher exact test was used to evaluate the association between clinical characteristics of the patients and MTC primary culture responsivity to everolimus in terms of cell viability reduction.

Results are expressed as the mean ± standard error of the mean (SEM). A Student’s paired or unpaired *t* test was used to evaluate the individual differences between means.

*P*-values < 0.05 were considered significant.

## Results

### Effects of everolimus on cell viability

To determine the effects of everolimus on cell viability of dispersed MTC cells, we assessed cell viability in MTC primary cultures after a 48 h treatment with 10 nM–1 μM everolimus. According to the extent of cell viability reduction recorded after treatment with everolimus, the primary cultures were divided in two groups: the cultures responding to any everolimus concentration with a significant (*P* < 0.05) cell viability reduction were considered as “everolimus-responders” (E-R), while those displaying a non-significant cell viability variation were considered as “everolimus-non-responders” (E-NR). According to this criterion, cultures from eight MTC were considered as E-R and 10 as E-NR.

Evaluating the patients clinical charts, we found that mean CT plasma levels of patients which primary cultures belonged to the E-NR group were ~fourfold higher than the mean CT plasma levels of patients which primary cultures belonged to the E-R group (*P* = 0.054). In addition, disease stage and inheritance were differently distributed among patients which primary cultures belonged to the E-R and those which primary cultures belonged to the E-NR group. In particular, E-NR MTCs were significantly associated with a disease stage ≥III (*P* < 0.02) and with sporadic disease (*P* < 0.03).

**Figure 1:**
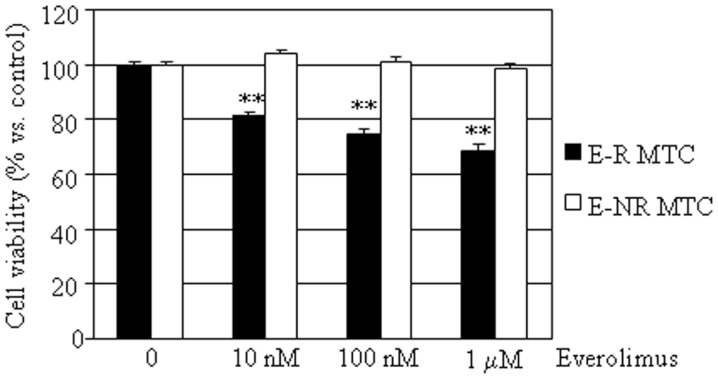
**Effects of everolimus on MTC primary culture cell viability**. MTC primary cultures were incubated in 96-well plates for 48 h in culture medium supplemented with everolimus at increasing concentrations from 10 nM to 1 μM, and control cells were treated with vehicle solution. Data from 18 MTC primary cultures were evaluated independently with six replicates each, and were expressed as the mean ± SEM percent cell viability vs. control cells. **P < 0.01 vs. control cells. As described in the Section “Results,” MTCs were divided according to cell viability inhibition after treatment with everolimus in E-R (eight samples, black bars) and E-NR (10 samples, white bars).

In the E-R group (black bars), everolimus dose-dependently reduced cell viability, from –19% at 10 nM to –31% vs. control at 1 μM (*P* < 0.01). In the E-NR MTCs (white bars), everolimus did not significantly modify cell viability (Figure 1). Further experiments were carried out only in the E-R group.

### Protein profiling

**Figure 2:**
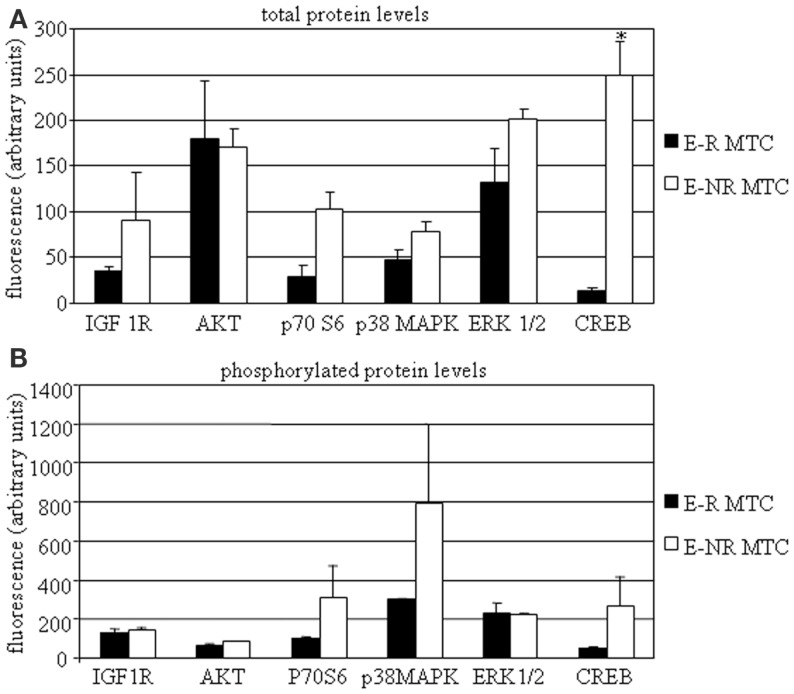
**MTC protein profiling**. MTC tissues were dissolved as described in the Section “Material and Methods” and assessed by Bio-plex^^®^^/Luminex^^®^^ Technology. Total **(A)** and phosphorylated **(B)** protein levels of IGF-I R, AKT, p70S6K, p38MAPK, ERK1/2, and CREB were assayed in E-R (black bars) and E-NR (white bars) samples. *P < 0.05 vs. E-R MTCs.

In a subset of MTC samples (including two E-R and four E-NR MTCs), we had a sufficient tissue amount that allowed us to investigate protein expression profile. We then assessed total and phosphorylated protein levels of IGF-I R, AKT, p70S6K, p38MAPK, ERK1/2, and CREB. We found that total IGF-I R, p70S6K, p38MAPK, ERK1/2, and CREB protein levels were higher in E-NR MTCs as compared to E-R MTCs (+153%, +259%, +68%, +53%, and +1735%, respectively). Statistical significance was observed for total CREB protein levels in E-NR MTCs vs. E-R MTCs (*P* < 0.02). On the contrary, total AKT protein levels were similar in the two groups (Figure 2A). In addition, phosphorylated IGF-I R, AKT, p70S6K, p38MAPK, and CREB protein levels were higher in E-NR MTCs as compared to E-R MTCs (+10%, +32%, +200%, +165%, and +426%, respectively). On the contrary, phosphorylated ERK1/2 protein levels were similar in the two groups (Figure 2B).

### Effects of IGF-I on cell viability in everolimus-responder MTCs

**Figure 3:**
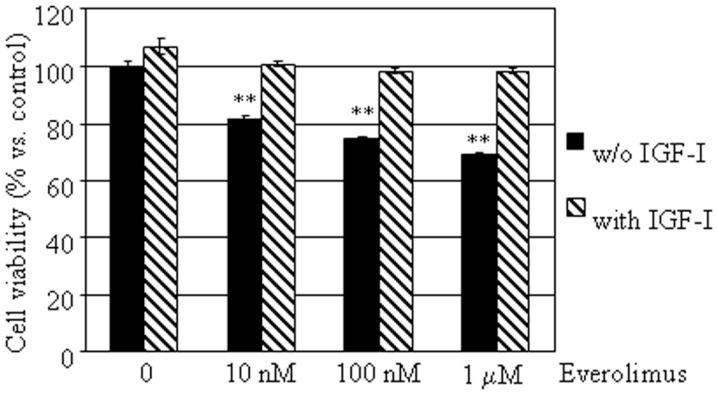
**Effects of IGF-I on MTC primary culture cell viability**. MTC primary cultures were incubated in 96-well plates for 48 h in culture medium supplemented with everolimus at increasing concentrations from 10 nM to 1 μM, and control cells were treated with vehicle solution. Data from 18 MTC primary cultures were evaluated independently with six replicates each, and were expressed as the mean ± SEM percent cell viability vs. control cells. **P < 0.01 vs. control cells. E-R MTC were incubated with (dashed bars) or without 50 nM IGF-I (black bars).

To investigate whether everolimus inhibitory effects might involve growth factor activated pathways, cell viability was assessed in E-R MTC cultures treated with or without 50 nM IGF-I, in the presence or in the absence of 10 nM–1 μM everolimus. IGF-I did not significantly affect cell viability, but counteracted the inhibitory effects of everolimus at all concentrations tested (Figure 3).

### Effects of IGF-I on CT secretion in everolimus-responder MTCs

**Figure 4:**
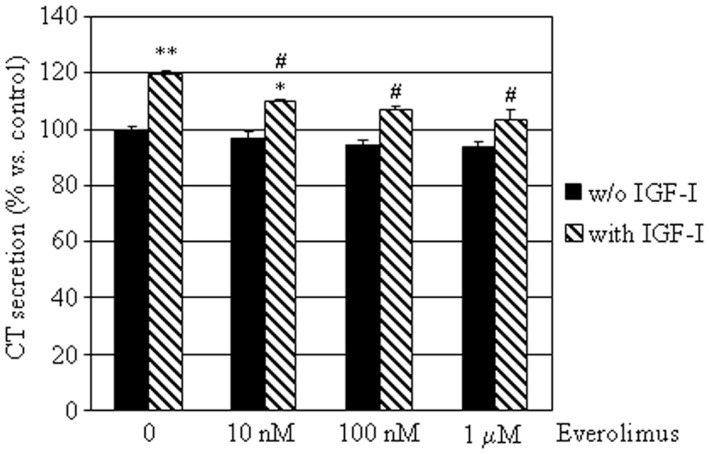
**Effects of everolimus and IGF-I on CT secretion by E-R MTCs**. E-R MTC primary cultures were incubated in 96-well plates for 6 h in culture medium supplemented with indicated substances. Control cells were treated with vehicle solution. CT secretion by each primary culture was then measured by ELISA. Data from eight E-R MTC primary cultures were evaluated independently with two replicates each and were expressed as the mean ± SEM percent CT secretion vs. control cells. *P < 0.05 and **P < 0.01 vs. control cells. ^#^*P* < 0.05 vs. IGF-I treated cells.

To determine the effects of everolimus on CT secretion by dispersed MTC cells, we assessed CT concentrations in conditioned medium from the eight E-R MTC cultures. CT levels were not significantly influenced by treatment with everolimus at any concentration tested. On the other hand, IGF-I significantly induced CT secretion (+19%; *P* < 0.01 vs. control), an effect that was reduced by co-incubation with everolimus at all concentrations tested (Figure 4).

## Discussion

This study demonstrates that IGF-I influences the effects of everolimus in a sub-group of human MTC primary cultures, supporting the hypothesis that IGF-I may hamper the effects of antiproliferative drugs in MTC. In addition, our results indicate that mTOR inhibitors may be effective in restraining cell proliferation only in a subset of MTCs, in keeping with previously reported *in vivo* data (13, 18). Indeed, Lim et al. show that only one out of the nine MTC patients treated with everolimus displays a ~20% reduction in tumor bulk, while the other patients show either stable or progressive disease (18). These data support the hypothesis that other survival pathways, including those activated by *RET* mutations, are active in MTC and may hamper the effects of mTOR inhibitors. The latter may be cytostatic, since it has been demonstrated that everolimus treatment of pancreatic neuroendocrine tumor (pNET) and MTC cell lines inhibits cell growth by increasing the G0/G1 phase of the cell cycle (12, 19). Moreover, it is widely demonstrated that mTOR inhibition has a significant antiproliferative effect on pNET cell lines (20) as well as on other tumor cells (8, 9).

Insulin-like growth factor I is confirmed as a protective growth factor toward C-cell survival, in keeping with previous evidence (21). Our data confirm that IGF-I does not stimulate cell proliferation of MTC primary cultures, but protects them from the effects of everolimus, suggesting that escape from therapy may occur in the presence of IGF-I or similar growth factors. This hypothesis is further strengthened by the finding that E-NR MTC display higher levels of IGF-I R and therefore may possibly be more sensitive to the protective effects of IGF-I. Indeed, we observed that IGF-I R displays higher phosphorylation levels in E-NR MTC as compared to E-R MTCs. This finding is in keeping with a greater activation of the IGF-I pathway, as also supported by higher phosphorylation levels (i.e., activation) of IGF-I downstream signaling proteins. Moreover, MTCs belonging to the E-NR group are significantly associated with sporadic and higher disease stage, indicating that this group represents a more aggressive disease.

In addition, we found that mTOR pathway is activated in MTC, as previously reported (22), especially in E-NR as compared to E-R MTCs. This finding indicates that mTOR signaling pathway over-activation is not invariably a marker of sensitivity to mTOR inhibitors in all endocrine and endocrine-related tumors (7, 9, 23). However, further studies are needed to identify a prognostic protein panel that may accurately predict responsivity of MTCs to mTOR inhibitors on clinical grounds.

Our results confirm that IGF-I promotes CT secretion in a subset of MTC primary cultures, as previously demonstrated (21). In addition, we found that this effect is hampered by everolimus, indicating that IGF-I modulates CT secretion involving the mTOR pathway, which, in turn, may regulate CT secretion only in response to secretory stimuli, such as IGF-I. It has been previously shown that IGF-I stimulates hormone secretion in parathyroid cells by modulating calcium channels (24) that are present in MTC cells (25). Calcium-dependent signaling pathways are induced by IGF-I through CREB phosphorylation (26) that, in turn, regulates CT secretion (27). Higher levels of both total and phosphorylated CREB protein were found in E-NR MTCs, that also display higher IGF-I R levels and higher CT plasma levels, supporting the hypothesis that IGF-I system may be over-activated and may promote CT secretion in E-NR MTCs. These tumors, despite displaying aggressive characteristics, may anyway benefit from treatment with everolimus, since this drug may control CT secretion. Indeed, as shown in the available clinical studies, everolimus treatment is effective in significantly reducing circulating CT plasma levels also when no effect on tumor bulk was apparent (13, 18, 28).

Our results also indicate that everolimus treatment may not result in both tumor growth control and hormone secretion reduction in all MTCs, as also previously found for treatment with somatostatin analogs, that could exert antiproliferative effects despite the lack of antisecretory activity and vice versa (14). These finding suggest that proliferation and secretion are controlled by two different mechanisms in human MTC.

In conclusion, our data show that IGF-I protects MTC primary cultures from the effects of everolimus and stimulates CT secretion by an mTOR mediated pathway that, in turn, may represent a therapeutic target in the treatment of aggressive MTCs. A synergistic effect of IGF-IR inhibitors and everolimus has already been hypothesized (29, 30). Indeed, mTOR inhibition increases Akt activity via the IGF-IR pathway, leading to the reduction of mTOR inhibitor effects (30). Therefore, IGF-IR signaling inhibition may prevent this positive feedback mechanism. The results of ongoing clinical trial employing both everolimus and the somatostatin receptor pan-agonist Pasireotide (SOM230 alone or in combination with RAD001 in patients with medullary thyroid cancer; NCT01625520) may help in clarifying this issue, also on the basis of the potent inhibitory effects of Pasireotide on IGF-I secretion.

## Author Contributions

EG: wrote the manuscript. CDP: performed protein expression profile. MR: performed primary cultures. FT: performed calcitonin secretion assay. TG: performed cell viability assay. RR: provided patients information. MP: provided patients information and surgical specimens. IMB: provided patients information and surgical specimens. EdU: supervised patients information and *in vitro* experiments. MCZ: supervised patients data and *in vitro* experiments, and wrote the manuscript.

## Conflict of Interest Statement

The authors declare that the research was conducted in the absence of any commercial or financial relationships that could be construed as a potential conflict of interest.
